# Belimumab and low-doses of mycophenolate mofetil as induction therapy of class IV lupus nephritis: case series and literature review

**DOI:** 10.1186/s12882-018-0847-z

**Published:** 2018-03-07

**Authors:** Domenico Paolo Emanuele Margiotta, Fabio Basta, Veronica Batani, Antonella Afeltra

**Affiliations:** Unit of Allergology, Clinical Immunology and Rheumatology, Department of Medicine, Università Campus Bio-Medico di Roma, via Álvaro del Portillo 21, 00128 Rome, Italy

**Keywords:** Belimumab, Mycophenolate mofetil, Lupus nephritis

## Abstract

**Background:**

The treatment of Lupus Nephritis (LN) is an unmet need in the management of patients with Systemic Lupus Erythematosus (SLE).

**Case presentation:**

We report two cases of women affected by Lupus Nephritis (LN) ISN/RNP Class IV with serological active disease, high disease activity and marked fatigue. In both cases, Mycophenolate mofetil (MMF), as induction therapy, was poorly tolerated because of gastrointestinal toxicity. Belimumab, together with low-doses of MMF, was effective as induction treatment leading to early achievement of complete renal response in these two selected cases of LN.

**Conclusions:**

We also report a literature review concerning the efficacy and safety of Belimumab in Lupus Nephritis. Further studies are needed to evaluate the use of Belimumab to manage the renal involvement in patients with Systemic Lupus Erythematosus, waiting for the results of ongoing randomized clinical trials.

## Background

Despite therapeutic advances, renal involvement still has a significant impact on prognosis and quality of life in patients with Systemic Lupus Erythematosus (SLE) [1]. A challenge in the management of LN is to achieve a prompt renal response, to maintain it, and avoid renal flares while preventing accrual of renal damage. It is also necessary to reach these targets while ensuring the patients the best possible quality of life and minimizing damage due to therapy. Nevertheless, currently, the renal response rate after 6 months is being achieved in less than one third of patients (in some studies, in less than 20% of patients) [2]. Mycophenolate mofetil (MMF) and cyclophosphamide (CYC) are both effective in the induction treatment of International Society of Nephrology/Renal Pathology Society (ISN/RPS) class IV LN, but often poorly tolerated [3]. Considering these efficacy and safety issues, defining the role of biologic therapies in LN treatment represents a main challenge. The contribution of Belimumab, a human antibody inhibiting the biological activity of B-cell activating factor (BAFF), in LN treatment is promising. Pooled data from phase 3 randomized clinical trials and several case reports indicate a possible efficacy of Belimumab in LN [4, 5]. We report two cases about the use of Belimumab in combination with low doses of MMF in the induction therapy of LN. We further analyse the available literature evidences about Belimumab and LN.

## Case presentation 

### Case 1

A 37-year-old woman, with an SLE disease duration of 10 years, has been followed-up in our Lupus Clinic since 2014. In 2004, she presented with ISN/RPS class III (A) LN and was treated with MMF 2 g/day until 2010 with complete renal response after 6 months of treatment. In subsequent years, she has not developed organ damage (last SLE damage index –SDI = 0). In the previous year, however, she manifested only mild constitutional symptoms (fatigue and superficial lymphadenopathy), peripheral arthralgia, and mild malar rash (British Isles Lupus Assessment Group – BILAG constitutional C, musculoskeletal C, mucocutaneous B). She presented with anti-double stranded DNA (anti-dsDNA) positivity (50 IU/ml with a cut-off of 9.9 IU/ml) and mild reduction of C3 complement fragment. Current treatment was hydroxychloroquine 400 mg/day, prednisone 5 mg/day, azathioprine 50 mg/twid. The patient no longer had signs of kidney disease (renal BILAG D).

**Fig. 1:**
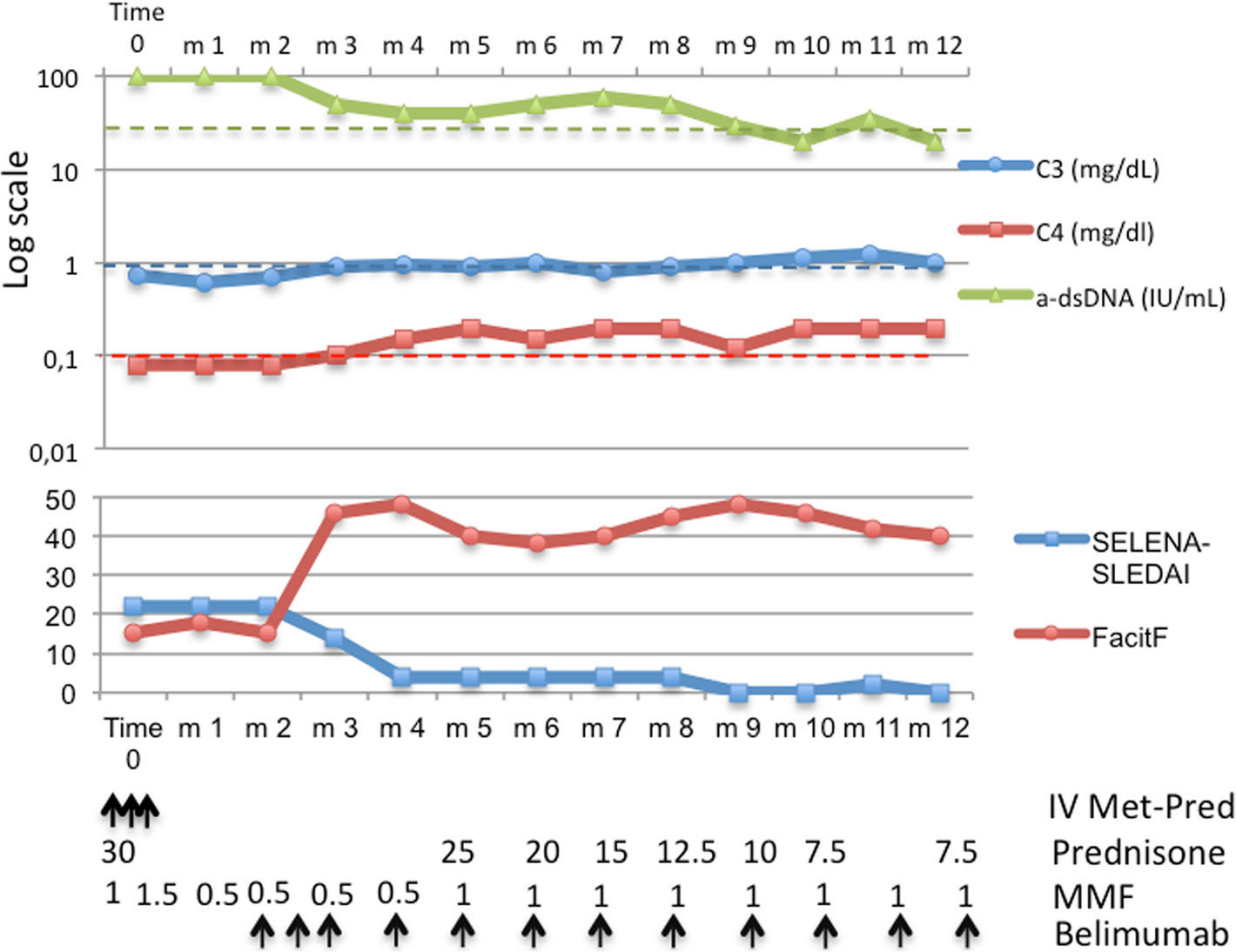
Serological activity, disease activity and fatigue in Case 1. IV Met-Pred, IV methylprednisolone at a dosage of 1000 mg/day for 3 days; arrow **↑** indicates IV drug infusion; prednisone, daily dosage of oral prednisone (mg/day); MMF, daily dosage of MMF (g/day); belimumab, IV infusion of belimumab

In 2014, routine follow-up tests demonstrated raised anti-dsDNA value (100 IU/ml), reduced complement fragment C3 0.74 g/L (reference range 0.9–1.8), C4 0.08 g/L (reference range 0.1–0.4). Urine analysis demonstrated the presence of 25 erythrocytes per high power field, 20 leucocytes per high power field and cellular casts. The 24-h proteinuria was 1500 mg. Renal function was preserved with normal value of creatinine and BUN. The patient had manifestation of neither nephritic nor nephrotic syndrome. The blood pressure profile was normal. The patient underwent renal biopsy. Histological examination showed ISN/RPS class IV-G (A) LN. Intravenous (IV) methylprednisolone at a dosage of 1000 mg/day for 3 days was started, followed by prednisone 30 mg/day (0.5 mg/Kg). MMF was initiated at a dose of 500 mg twice daily, and the dose was increased to 750 mg twice daily at week 2 and advanced weekly with the goal to reach the target dosage of 1000 mg three times daily. At week 4, the patient presented with persistent diarrhoea and mucorrhea. MMF was then tapered to 500 mg/day with prompt resolution of gastrointestinal symptoms but for persisting proteinuria (1300 mg/24 h) and active urinary sediment. At week 8, IV Belimumab 10 mg/Kg was introduced in combination with MMF 500 mg/day. The dose of MMF was gradually increased up to 1000 mg/day. After the first dose of the maintenance cycle of Belimumab, a complete renal response (according to LUNR trial) was achieved [6] and disease activity was reduced (SELENA SLEDAI from 22 to 4). Furthermore, fatigue promptly improved (increase of FACIT-Fatigue from 15 to 48) at the end of induction doses of Belimumab. After the third month of therapy, prednisone was tapered to 7.5 mg/day. After 2 years, the patient is still in complete renal response, with SELENA SLEDAI below 4 and high values of FACIT-Fatigue (Fig. 1).

### Case 2

**Fig. 2:**
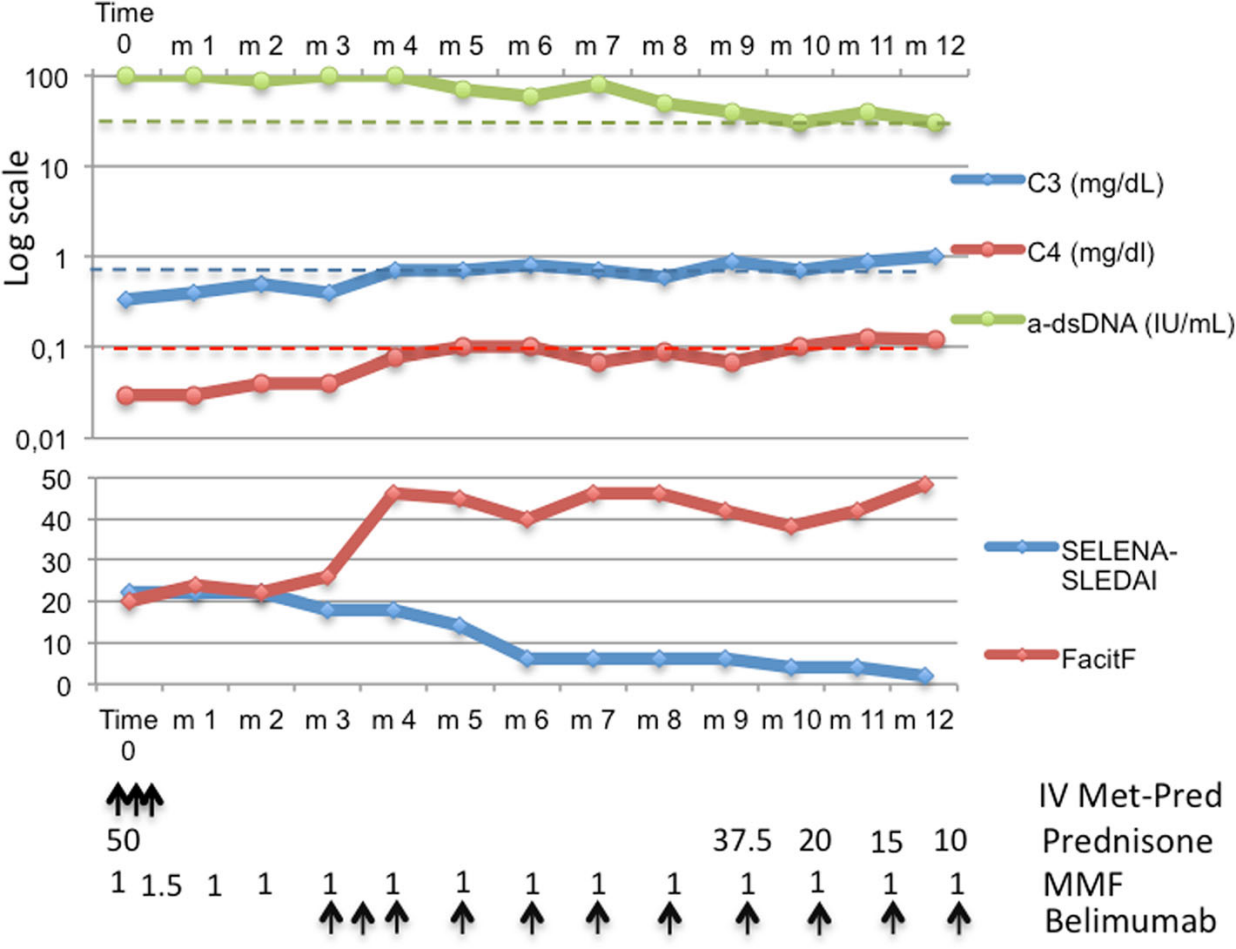
Serological activity, disease activity and fatigue in Case 2. IV Met-Pred, IV methylprednisolone at a dosage of 1000 mg/day for 3 days; arrow **↑** indicates IV drug infusion; prednisone, daily dosage of oral prednisone (mg/day); MMF, daily dosage of MMF (g/day); belimumab, IV infusion of belimumab

We report the case of a 35-year-old female patient. SLE was diagnosed in 2013. The first disease manifestation was nephrotic syndrome. The patient underwent renal biopsy with evidences of ISN/RPS class IV-G (A) LN. She was initially treated in another hospital with a low-dose IV CYC regimen (6 fortnightly pulses at a fixed dose of 500 mg) according to EURO-LUPUS protocol followed by azathioprine 50 mg/twid [7]. After 3, 6 and 12 months, no renal response was achieved. The patient has been followed in our Lupus Clinic since the end of 2014. She manifested constitutional symptoms (low degree fever and fatigue), mucocutaneus symptoms (malar rash and oral aphthous ulceration), low complement fragment C3 0.34. mg/dL (reference range 0.9–1.8) and C4 0.03 mg/dL (reference range 0.1–0.4) and anti-dsDNA positivity (100 UI/ml). Her 24-h proteinuria was 4400 mg, creatinine was 1.1 and the urine analysis showed 20 erythrocytes per high power field and 10 leucocytes per high power field. The disease activity according BILAG was: constitutional C, musculoskeletal D, mucocutaneous B, renal A. The patient was considered refractory to the therapy. IV methylprednisolone at a dosage of 1000 mg/day for 3 days was started, followed by prednisone 50 mg/day (about 1 mg/Kg). The patient was treated with MMF at a dose of 500 mg/twid, gradually increased to 1000 mg/twid. After a month, the patient presented with epigastralgia and persistent aqueous diarrhoea. The MMF dosage was reduced to the maximum tolerated dose of 1000 mg/day. At the end of the second month of therapy, no renal response was achieved. We decided to introduce combination therapy of IV Belimumab 10 mg/Kg with a low dose of MMF (1000 mg/day) and prednisone 1 mg/Kg/day. After 3 months of combination therapy, complete renal response was achieved and prednisone therapy was tapered to 10 mg/day. At the end of the induction cycle, we noticed a strong improvement of fatigue (FACIT-Fatigue from 20 to 48). After 2 years of starting therapy with Belimumab, a complete renal response was maintained with SELENA-SLEDAI below 6 (Fig. 2).

## Discussion

**Fig. 3:**
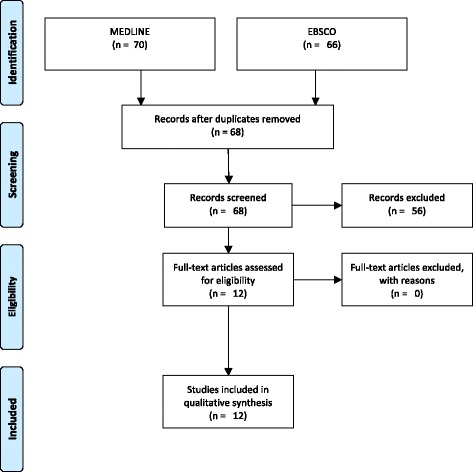
PRISMA flow-diagram

We performed a literature search, from year 2000 to present, in the PubMed and EBSCO databases using the following MeSH terms: (“belimumab”[Supplementary Concept] OR “belimumab”[All Fields]) AND (“lupus nephritis”[MeSH Terms] OR (“lupus”[All Fields] AND “nephritis”[All Fields]) OR “lupus nephritis”[All Fields]). We also performed the search using free terms: “belimumab” AND “lupus nephritis”. We report the PRISMA flow-diagram in Fig. 3. Of the initial 70 records, we selected only original works, letters to the editor or case report concerning the use of Belimumab in LN. Exclusion criteria were: review, full text not available and papers not in English. After selection, 12 articles were included in the review.

We included in the literature review the following articles: 9 case reports, 1 prospective cohort study, 1 article reporting pooled data from two phase III randomized clinical trials and 1 systematic review.

### Pooled data from randomized clinical trials

Data from phase II and III clinical trials showed that Belimumab is able to achieve SLE responder index (SRI) after 1 year of treatment more frequently than the standard of care. Moreover, Belimumab is able to reduce disease activity and prevent severe disease flare, also displaying steroid-sparing ability [5, 8–11]. In a subanalysis of phase III clinical trials BLISS 52 and 76, 16% of patients had renal involvement according to SELENA-SLEDAI, 10.6% had a renal BILAG A or B score and 20.4% had a 24-h proteinuria 500 mg. Considering patients with baseline 24-h proteinuria ≥1000 mg, at week 52, renal remission was observed in 70.5% of patients treated with belimumab 10 mg/Kg and in 58.7% of placebo, and the median time to 1st renal remission was shorter in the belimumab group. In the overall pooled population, patients treated with belimumab 10 mg/Kg presented fewer renal flares (1.4%) compared to placebo (3%). Considering both patients with baseline 24-h proteinuria > 200 mg (640 patients) and > 1000 mg (218 patients), those treated with belimumab 10 mg/Kg presented significantly greater median reduction in proteinuria during weeks 12–52 than those receiving placebo. Fifty-six of 267 patients with SELENA-SLEDAI renal involvement and 29 with renal BILAG A or B at baseline were treated with combination therapy of belimumab and MMF. At week 52, Renal improvement was seen in 63.2% of patients in 10 mg/Kg belimumab group compared to 27.8% of placebo. The proportion of patients who developed renal flare was lower in patients randomized to receive 10 mg/Kg belimumab (1.5%) than placebo (4.9%). The improvement in proteinuria among patients treated with belimumab plus MMF was not significant [4].

### Prospective cohort study

Iaccarino et al. reported results from an Italian prospective cohort of 67 SLE patients treated with belimumab added to background therapy. The mean follow-up was 16.2 ± 9.5 months. Overall, disease activity (mean SLEDAI-2 K) and mean prednisone daily dose decreased during the treatment. Moreover, authors observed a reduction in the lupus flare rate 1 and 2 years after belimumab initiation compared to the period before. Sixteen (23.9%) patients presented with refractory LN at baseline. Belimumab was started in 10 of these patients due to persistent 24-h proteinuria> 1000 mg after at least 1 year from the start of the initial therapy, and in the remaining 6 patients because of a mild renal flare during the subsequent therapy. Considering only the 16 patients with LN, after 18 months of belimumab treatment, mean 24-h proteinuria significantly decreased from baseline (1.27 ± 0.68 g vs 0.69 ± 0.71 g). Belimumab was well tolerated and only 2 adverse events, a deep vein thrombosis and a pneumonitis, were observed during follow-up [12].

### Systemati review

Sciascia et al. reported a systematic review of the evidences of belimumab efficacy on renal outcomes. They included in the analysis only studies reporting the effect on renal parameters from Ovid MEDLINE and from the abstract of EULAR and ACR/ARHP Annual Meetings (2011–2015). A total of 2004 patients with SLE were identified from the 11 studies that included 234 of those who had LN and received belimumab. Thirteen patients out of 234 (5.5%) received belimumab for active LN. One hundred twenty-nine (55.1%) of the 234 patients with LN at baseline showed an improvement in renal parameters after treatment with belimumab. The therapy with belimumab was able to reduce proteinuria of a median factor of 38% in patients with baseline proteinuria > 0.2/g/day. Moreover, a rate of renal response of 70.7% was observed in patients with baseline proteinuria ≥1 g/day. In a mean observation time of 1.1 years, the authors reported a low rate of annual renal flare (1.7%) [13].

### Case reports

The demographic and SLE disease features of patients included in case reports are described in Table 1.

**Table 1:** Description of studies included in Literature Review

Author, Year	Type of Study	Subjects	Baseline Disease features	Baseline Nephritis	Previous Therapy	Treatment	Outcome	Results
Staveri, 2016 [20]	Case series	1 caucasian greek female 31 yrs. old	Seropositive Active lupus (constitutional, mucocutaneous, hematologic involvement, arthritis,)	No baseline kidney involvement	Oral CG, HCQ, MTX, RTX and AZA IV GC pulses	After 3 months of treatment with Belimumab, onset of Proteinuria 1600 mg/24 h. Diagnosis of LN WHO Class III. Treated with AZA 3 mk/Kg		
		1 caucasian greek female 38 yrs. old	Seropositive Active lupus (fever, arthritis, pleuropericarditis, NPS involvement)	No baseline kidney involvement	Oral GCs and MTX CNS lupus: CYC e and RTX	After 3 months of treatment with Belimumab, active urinary sediment, proteinuria (6 g/24 h). Diagnosis of LN WHO Class V. Treated with MMF 2 g/day		
Danve, 2016 [19]	Case Report	1 caucasian female 38 yrs. old Pregnancy planning	Seropositive Active lupus and aPL syndrome (mucocutaneous lupus, angioedema, lupus nephritis, leukopenia, anti-phospholipid syndrome)	Active urinary sediment No biopsy proven	Before pregnancy: Oral GC, HCQ, AZA, RTX	Ongoing: HCQ Before Pregnancy: Belimumab + MMF for 6 months than Belimumab alone During pregnancy: Belimumab till the 32 week During breast-feading: Belimumab was resumed 2 weeks after delivery	Serum creatinine, UPC ratio	Clinical remission Before pregnancy, during and after pregnancy
De Scheerder, 2016 [16]	Case Report	1 African female 26 yrs. old	Chronic dacryoadenitis Seropositive Active lupus (mucocutaneous, NPS, ocular vasculitis)	LN Class V LN Class V Proteinuria 2.72 g/24 h SELENA-SLEDAI 24	GCs, HCQ, MMF 3 g/day, After 2 months MMF was tapered to 1.5 g/day and tacrolimus was associated	Belimumab in combination with MMF 1.5 g/day, Tacrolimus, GCs and HCQ	SELENA-SLEDAI BILAG Proteinuria	Proteinuria was 1.93 g/24 h after 1 month, 0.19 g/24 h after 3 months and 0.07 g/24 h after 6 months
Furer, 2016 [22]	Case series	1 female 25 yrs. old	Seropositive Active SLE (mucocutaneous, hematologic involvement, arthritis)	No baseline LN	HCQ, AZA, MT	Belimumab monotherapy was added with a favourable clinical response. After 2 years, the treatment was discontinued (urticaria). Eight months after belimumab discontinuation severe flare with new-onset LN class IV		
Gonzalez-Echavarri, 2016 [17]	Case report	1 female 25 yrs. old	Seropositive Longstanding Active SLE with class IV LN, arthritis, constitutional and vasculitic mucocutaneous involvement	LN Class IV	LN induction with CYC and maintenance with AZA. Several LN flares: different therapies, including 4 courses of RTX. After 1 year new renal flare unresponsive to GCs, IVIG, CYC	Belimumab + MMF 750 mg/day, Tacrolimus 7 mg/day, Prednisone 5 mg/day, HCQ	Proteinuria	Proteinuria started to decrease at month 2 with clinical remission at month 4 (0.1 g/24 h)
Simonetta, 2016 [18]	Case report	1 Bolivian female 23 yrs. old	Seropositive Active SLE (constitutional, mucocutaneous, serositic, hematologic involvement, arthritis, nephritis)	LN Class IV S(A) UCP ratio > 1.5 SELENA-SLEDAI> 20	GCs, HCQ MMF 2.5 g/day Belimumab + MMF RTX 1 g × 2	Second course of Belimumab after Rituximab	SELENA SLEDAI UPC ratio	SELENA-SLEDAI 0 Renal Response UPC ratio < 0.5 Remission of systemic manifestations
Kraaij, 2014 [15]	Case series	1 female 32 yrs. old	Seropositive Active SLE (constitutional, mucocutaneous involvement, nephritis)	LN Class IV	MMF, Eurolupus CYC and again MMF with no renal response. RTX followed by MMF with partial response	Belimumab monotherapy	SELENA SLEDAI Proteinuria	Proteinuria decreased to 0.9 g/24 h, SELENA-SLEDAI 6
		1 male 42 yrs. old	Seropositive Active SLE (constitutional, mucocutaneous and NPS involvement, nephritis)	LN Class IV	LN induction with CYC and MMF, each followed by MMF, GC, HCQ maintenance. Renal flare treated with RTX followed by MMF with initial partial remission and relapse after MMF withdrawal	Belimumab monotherapy	SELENA SLEDAI Proteinuria	Proteinuria improved (1.5 g/24 h), SELENA-SLEDAI 4
Fliesser, 2013 [14]	Case Report	1 female 19 yrs. old	Seropositive Active SLE (constitutional, serositic, mucocutaneous, haematologic involvement, nephritis)	LN Class III (A/C)	HCQ, MMF, GC with no renal response	Belimumab in combination with MMF (2 g/day tapered to 1 g/day), GCs and HCQ	Proteinuria	Progressive decline of proteinuria (409 mg/24 h after 2 weeks; 202 mg/24 h after 4 weeks; 1 year later 75 mg/d24h and sediment normalization
Sjowall, 2014 [21]	Case report	1 caucasian female 62 yrs. old	Seropositive Active Lupus and aPL syndrome (erosive arthritis, serositic involvement) History of cervical cancer in situ and ocular melanoma	No baseline kidney involvement	Oral GC, HCQ, AZA, MMF	Belimumab in combination with MMF 1 g/day. After 3 months: remission of constitutional and serositic involvement, beginning of steroid spare. After 10 months, recurrence of pleural effusion and onset of LN class III. Treated with CYC (EuroLupus).		

*yrs* years; *LN* Lupus Nephritis, *Oral CG* oral glucocorticoids, *HCQ* hydroxycloroquine, *MTX* methotrexate, *RTX* rituximab, *MMF* mycophenolate mofetil, *AZA* azathioprine, *IV GC pulses* intravenous glucocorticoids, *CYC* cyclophosphamide, *mg/24 h* milligrams /24 h, *UPC ratio* Urine Protein to Creatinine Ratio, *CNS lupus* Central Nervous System Lupus, *aPL* anti-phospholipids, *NPS* neuro-psychiatric

### Case reports with favourable outcomes (LN refractory to MMF or CYC)

In 2013, Fliesser et al. reported the case of a young woman with active class III (A/C) LN, constitutional and muco-cutaneous involvement. LN occurred a few months after SLE diagnosis with 24-h proteinuria up to 1400 mg and nephritic urinary sediment, despite baseline therapy of MMF 2 g/day, hydroxycloroquine 300 mg/day and prednisolone 25 mg/day. About 1 month before belimumab, MMF dose had been increased to 3 g/day. Then, the patient received a steroid pulse (total dose of 2.5 g of metilprednisolone over 3 days) and belimumab was added to baseline therapy. Authors described a rapid improvement in proteinuria with a fall to 400 mg/day after 2 weeks and to 200 mg/day after 1 month. A year later, the patient was in clinical remission with belimumab and MMF 1 g/day [14].

Kraaij et al. described two cases of refractory class IV-S(A) and -G(A) LN. The first patient, a 32-year-old woman with renal, constitutional and muco-cutaneous involvement, received two induction regimens (MMF and CYC, Euro-Lupus protocol) and then rituximab followed by maintenance with MMF with partial reduction in proteinuria. Then, MMF was discontinued due to intractable nausea and weight loss. Belimumab was commenced in monotherapy 7 months after rituximab. After 18 months, proteinuria remained below 1 g/day. The second patient was a 42-year-old man with constitutional, muco-cutaneous and neuro-psychiatric manifestations. He was treated with two induction regimens (CYC and MMF) and with MMF as maintenance without renal response. Partial renal response was obtained with rituximab followed by MMF. However, the patient was not able to adhere to MMF therapy because of gastrointestinal intolerance, leading to renal flare. Then, the patient was treated with belimumab and prednisolone. After 12 months, the patient was in low disease activity status and prednisolone was tapered to zero [15].

The case reported by De Scheerder et al. concern a 26-year-old African female with ocular vasculitis, mucocutaneous, central nervous system involvement and class V LN. The initial therapy with MMF up to 3 g/day was tapered to 0.5 mg/day and associated with tacrolimus because of persistent proteinuria and ocular vasculitis. After 1 month, belimumab was added with rapid and progressive decrease of proteinuria and amelioration of ocular vasculitis. After 6 months, complete renal response was reached. After 1 year, therapy with MMF and tacrolimus was tapered until complete withdrawal. After 2 years, the therapy with glucocorticoid was stopped with the maintenance of long-term complete remission [16].

### Case reports with favourable outcomes (LN refractory to rituximab)

In 2016, Gonzalez-Echavarri et al. reported the case of a 25-year-old woman with longstanding relapsing class IV-G(A) and than -G(A/C) despite several therapeutic regimens, including CYC, MMF, azathioprine, combination of MMF and tacrolimus, rituximab in association with MMF or CYC. Belimumab was introduced in combination with prednisone, hydroxycloroquine, MMF and tacrolimus leading to complete remission after 4 months. The remission was maintained after 2 years and tacrolimus was stopped [17].

Simonetta et al. described the case of a 23-year-old woman with seropositive lupus and mucocutaneous, articular, serositic and hematologic involvement. Kidney biopsy proved Class IV-S(A) LN which was treated with high doses of glucocorticoids and MMF 2.5 g/day without renal or systemic response. After 6 months, Belimumab was added to therapy with an initial transient improvement in proteinuria. After a disease flare, Belimumab was stopped and Rituximab 1000 mg 2 week apart was administered leading to serologic improvement but not to renal response. Authors decided to retreat the patient with Belimumab obtaining sustained renal response and remission of systemic manifestations [18].

### Case reports with favourable outcomes (LN during pregnancy)

The possible helpfulness of Belimumab in the treatment of lupus nephritis in a pregnancy planning setting was described in the case reported by Danve et al. The authors reported the case of a young woman with SLE and anti-phospholipid syndrome complicated by lupus nephritis. The patient was treated with MMF and prednisone. To allow to conceive, MMF was discontinued. The patient was treated with azathioprine and then Rituxmab, but both were/had been withdrawn because of safety issues. The authors decided to start belimumab plus MMF for 6 months and then belimumab alone till the 32nd week of pregnancy. The patient remained in remission throughout the pregnancy and delivered at term a female baby with mild Ebstein’s anomaly (mild displacement of tricuspid valve with mild to moderate regurgitation) on ECHO. Belimumab was resumed during breastfeeding [19].

### Case reports with adverse outcomes

Staveri et al. described two cases of active SLE without renal involvement. In both patients, Belimumab was prescribed to treat refractory arthritis, mucocutaneous, constitutional and hematologic involvement. After a short course of therapy, the patients developed class III and class V LN. In both patients, Belimumab was discontinued and therapy with, respectively, high doses of AZA and MMF was initiated [20].

Sjowall et al. reported the case of an initially mild SLE. The clinical setting worsened with development of recurrent serositic involvement. The patient was seropositive and clinically active and fitted well in the subgroup of patients who should benefit from belimumab. However, after an initial improvement together with a steroid-sparing effect, the patient developed class III LN during Belimumab and low-dose MMF combination therapy [21].

Furer et al. reported three cases of SLE flare after belimumab cessation registered by members of Israeli Society of Rheumatology. Among the case series, a young woman, treated with belimumab for 2 years, developed a severe lupus flare with new onset of class IV LN 8 months after belimumab discontinuation. The authors hypothesised a possible rebound effect due to BAFF levels increasing after belimumab cessation, but this speculation was not proved [22].

## Conclusions

We reported two cases of patients affected by Class IV LN unresponsive or not tolerant to conventional therapy. In both cases, the MMF target dosage of 1000 mg 3 times a day was not achieved because of gastrointestinal symptoms. Gastrointestinal involvement is the most frequent adverse event causing MMF discontinuation (with prevalence rates close to 30%) and responds to dose reduction. In both cases, we decided to maintain MMF at low doses in combination with Belimumab. The interest of our case is the use of Belimumab in combination with MMF in induction therapy of LN, allowing to keep low doses of MMF. This treatment strategy could be a useful option in patients with intolerance to MMF. As demonstrated in the second case, Belimumab plus low doses of MMF may also be effective in LN refractory to CYC.

Moreover, Belimumab has additional therapeutic values: as clearly demonstrated in phase III trial, Belimumab ensures a better control of fatigue and quality of life, and displays a steroid sparing capacity, in comparison with standard therapy. This advantage in improving quality of life was a relevant aspect described in our cases.

The majority of the studies included in the present literature review demonstrated the efficacy and safety of Belimumab in the treatment of LN. In the pooled data from the phase III clinical trial BLISS 52 and 76, the treatment with belimumab increased the rate of renal remission and reduced the time to reach this outcome. Moreover, in patients treated with belimumab, a reduced incidence of renal flares and a decrease in proteinuria were observed [4]. Similar findings concerning the efficacy in proteinuria reduction were reported by the Italian multicentre prospective study [12]. The interest of the systematic review by Sciascia et al. is the analysis of studies presented as abstract at EULAR or ACR meetings, increasing the number of patients with LN treated with Belimumab up to 234. The authors confirmed the efficacy and safety of belimumab in LN [13].

We included in the present review all case reports concerning belimumab and LN. In these reports, four SLE patients were successfully treated with belimumab for class III, IV or V LN after failure of cyclophosphamide or MMF. In three of these cases, the treatment with belimumab led to reaching and maintaining disease remission [14–16]. Furthermore, belimumab was able to induce systemic and renal remission in two patients unresponsive to Rituximab, with one of them presenting a longstanding multi-drug refractory SLE [17, 18]. The adjunctive concern of these case reports, in comparison to data from large cohort studies, especially phase III clinical trials, is the observation of belimumab efficacy in refractory LN and, often, in challenging clinical settings, characterized by longstanding disease and inefficacy or intolerance to several treatments.

In our literature review, we also took into account the reports of LN developed during belimumab treatment or after therapy withdrawal, in patients without baseline LN [20–22]. Considering the available data, it is impossible to speculate if belimumab had a facilitative effect on LN development or if belimumab was not able to contrast disease progression in these cases. Nevertheless, these cases are isolated reports; it seems reasonable to monitor renal function during treatment with belimumab and after belimumab withdrawal according to current clinical practice and available SLE guidelines.

In conclusion, our case reports, in concert with available literature evidences, suggest that belimumab could be an effective and safe option to treat LN, even in refractory cases, allowing to spare glucocorticoids and immunosuppressants, such as MMF. Further studies are, however, necessary to confirm our results, while we are waiting for the ongoing randomized clinical trials on the use of belimumab in LN.

## Abbreviations

aPL syndrome: anti-phospholipids syndrome
AZA: Azathioprine
BILAG: British Isles Lupus Assessment Group
CYC: Cyclophosphamide
GC: Glucocorticoids
HCQ: Hydroxycloroquine
IV: Intravenous
IVIG: IV Immunoglobulin
LN: Lupus Nephritis
MMF: Mycophenolate mofetil
MTX: Metotherxate
NPS: Neuro-psychiatric
RTX: Rituximab
SLE: Systemic Lupus Erythematosus
UPC ratio: Urine protein creatinine ratio
yrs: years
